# Effects of Acute Dietary Nitrate Supplementation on Selected Isokinetic Performance Variables in Physically Active Men

**DOI:** 10.3390/nu18142377

**Published:** 2026-07-21

**Authors:** Jakub Jurga, Jarosław Kabaciński, Anna Fryzowicz, Michał Murawa

**Affiliations:** 1 Doctoral School, Poznan University of Physical Education, 61-871 Poznan, Poland; jurga@awf.poznan.pl; 2 Department of Biomechanics, Poznan University of Physical Education, 61-871 Poznan, Poland; kabacinski@awf.poznan.pl (J.K.); fryzowicz@awf.poznan.pl (A.F.)

**Keywords:** dietary nitrate, beetroot juice, isokinetic strength, torque, muscle power, electromyography, nitric oxide, maximum voluntary contraction, fatigue

## Abstract

Background: Dietary nitrate may enhance skeletal muscle performance by increasing nitric oxide bioavailability. Although its benefits for endurance exercise are well documented, less is known about its effects on isokinetic strength and power performance. This study examined the effects of acute nitrate ingestion on isokinetic performance, neuromuscular activity, and metabolic responses in physically active men. Methods: Thirteen physically active young men (25.0 ± 5.2 years) participated in a randomized, double-blind, placebo-controlled crossover study. During separate experimental sessions, participants consumed either nitrate-rich beetroot juice containing 800 mg of nitrate or a low-nitrate placebo. Following a 2 h absorption period, they performed isokinetic knee extension–flexion tests at angular velocities of 60°/s, 180°/s, and 240°/s. Surface electromyography was recorded from the vastus medialis oblique and vastus lateralis oblique muscles, and blood lactate concentrations were measured before and after exercise. Results: Nitrate supplementation significantly improved peak torque, relative peak power, and selected torque angle variables at 60°/s and 180°/s compared with placebo (*p* < 0.05). In contrast, no significant differences were observed during the 240°/s fatigue protocol (*p* > 0.05). No significant differences were observed in electromyographic parameters or post-exercise blood lactate concentration. Similarly, post-exercise blood lactate concentrations were comparable between treatments (*p* > 0.05). Conclusions: Acute dietary nitrate supplementation may improve selected isokinetic performance variables during low- and moderate-velocity contractions, whereas no significant effects were observed during the 240°/s fatigue protocol. No changes were detected in electromyographic parameters or post-exercise blood lactate concentration. The physiological mechanisms underlying these findings remain to be established.

## 1. Introduction

Nitric oxide (NO) is a key signalling molecule involved in the regulation of numerous physiological processes, including vascular tone, mitochondrial respiration, and skeletal muscle contractility. Traditionally, nitric oxide has been produced endogenously through the oxidation of L-arginine by nitric oxide synthase (NOS) [1]. However, an alternative pathway involving the sequential reduction of dietary nitrate (NO_3_^−^) to nitrite (NO_2_^−^) and subsequently to nitric oxide has been described [2]. This nitrate–nitrite–NO pathway becomes particularly active under conditions of reduced oxygen availability and lower pH, such as those occurring during intense muscular exercise, thereby increasing NO bioavailability in working skeletal muscle [3].

Because of these physiological mechanisms, dietary nitrate supplementation has received considerable attention as a potential ergogenic aid. Nitrate-rich foods, particularly beetroot juice, have been widely investigated in relation to exercise performance. Early studies demonstrated that dietary nitrate supplementation can reduce the oxygen cost of submaximal exercise and improve mitochondrial efficiency [4,5]. In addition, improvements in endurance performance, including increased time to exhaustion and improved time trial performance, have been reported in both recreationally active individuals and trained athletes [6,7]. These ergogenic effects are thought to be mediated through improved mitochondrial efficiency, enhanced muscle perfusion, and altered muscle contractile function [6].

Although most studies have focused on endurance exercise, emerging evidence suggests that nitrate supplementation may also influence neuromuscular performance variables such as muscle power and contraction velocity. Coggan et al. [8] demonstrated that acute nitrate supplementation increased maximal contraction velocity and peak power of knee extensor muscles during isokinetic dynamometry. More recently, Daneshparvar et al. [9] reported that acute beetroot juice supplementation improved lower-body isokinetic and isometric strength, average power, and muscular endurance in trained climbers. Eroglu et al. [10] demonstrated that acute beetroot juice supplementation enhanced peak and mean power during a 30 s Wingate test in trained football players, further supporting the potential ergogenic effects of dietary nitrate during short-duration, high-intensity exercise. These findings provide further evidence that the ergogenic effects of dietary nitrate may extend beyond endurance exercise to selected measures of neuromuscular. Previous studies suggest that nitrate supplementation may enhance muscle performance during high-velocity contractions, potentially owing to the preferential influence of nitric oxide on fast-twitch (type II) muscle fibres [11]. However, the available evidence remains inconsistent. While Daneshparvar et al. [9] reported improvements in selected strength-related outcomes following acute beetroot juice supplementation, Rodrigues et al. [12] found no significant benefits during high-intensity resistance exercise, highlighting the need for further investigation under different exercise conditions. Because force production, contraction velocity, muscle power, and fatigue characteristics differ substantially across isokinetic testing performed at different angular velocities, evaluating performance under multiple testing conditions may provide a more comprehensive understanding of the ergogenic effects of dietary nitrate.

Despite these findings, relatively few studies have investigated the effects of nitrate supplementation on muscle performance during strength-oriented protocols such as isokinetic testing. Moreover, limited research has examined neuromuscular activation patterns assessed using surface electromyography (EMG) in this context. Consequently, it remains unclear whether improvements in isokinetic performance following nitrate supplementation are accompanied by alterations in neuromuscular activation or primarily reflect peripheral adaptations. Evaluating EMG responses may provide insight into whether potential performance improvements are associated with altered motor unit recruitment or improved neuromuscular efficiency.

In addition, the metabolic response to high-intensity exercise, commonly assessed using blood lactate concentration, remains insufficiently explored in relation to nitrate supplementation during strength and fatigue protocols. Assessment of post-exercise blood lactate may also help determine whether any ergogenic effects are accompanied by alterations in the metabolic response to maximal isokinetic exercise. Therefore, the aim of this study was to examine the effects of acute ingestion of a nitrate-rich supplement compared with placebo on isokinetic hamstrings and quadriceps performance as well as neuromuscular activity of the vastus medialis oblique and vastus lateralis oblique muscles.

## 2. Materials and Methods

### 2.1. Participants

The sample size was estimated a priori using a two-way repeated-measures analysis of variance in Dell Statistica 13.1 software. The calculation was based on mean and standard deviation values of plasma nitrate/nitrite (NOx) concentrations obtained in our previous study [13] and supported by previous investigations of the physiological response to acute dietary nitrate supplementation [14]. Plasma NOx was selected because the intervention was designed to induce an increase in nitric oxide bioavailability, which constitutes the primary physiological response to dietary nitrate ingestion and the proposed mechanism underlying subsequent changes in muscle performance. The significance level (α) and statistical power (1 − β) were set at 0.05 and 0.95, respectively. Based on these assumptions, the minimum required sample size was estimated to be 10 participants.

Thirteen physically active men (age: 25.0 ± 5.2 years; body mass: 82.2 ± 11.1 kg; height: 183 ± 5 cm) volunteered to participate in this study. All participants reported their right lower extremity as their dominant limb and engaged in regular recreational physical training at least four times per week and represented a variety of sporting backgrounds. Inclusion criteria were age ≥18 years, participation in at least three structured training sessions per week, and no musculoskeletal injuries in the previous 12 months. Participants were excluded if they smoked or had any condition or injury within the past year that could affect neuromuscular performance. Participants were informed about all experimental procedures, potential risks, and benefits prior to participation and provided written informed consent. The study protocol was approved by Bioethics Committee at Poznan Medical University (approval number: 239/25; 3 April 2025) and was conducted in accordance with the principles outlined in the Declaration of Helsinki. The protocol was registered in ClinicalTrials.gov (NCT07605455; 26 May 2026).

### 2.2. Study Design

**Figure 1:**
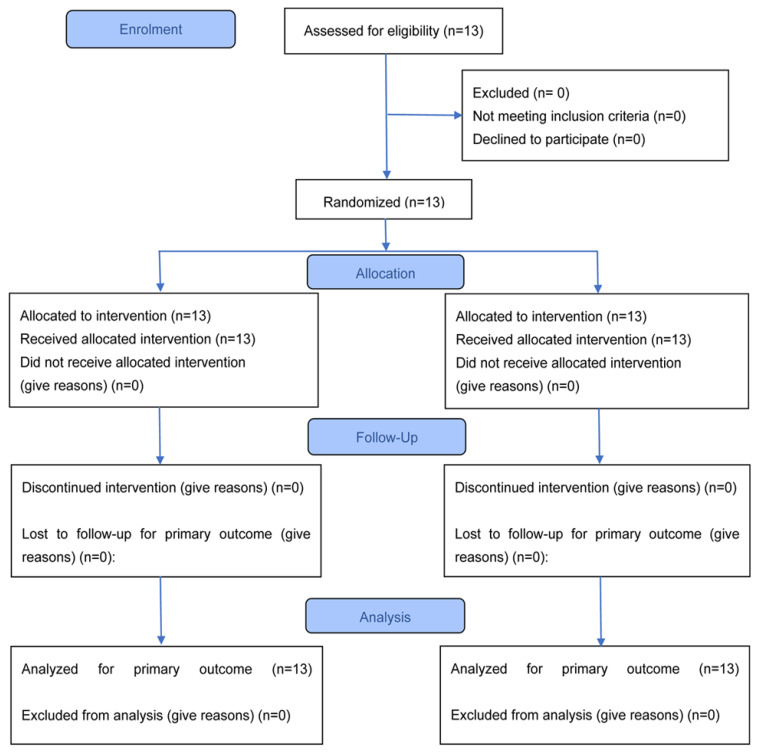
CONSORT 2025 Flow Diagram. A flow diagram of the progress through the phases of a randomized trial of two groups (enrolment, intervention allocation, follow-up, and data analysis).

A randomized, double-blind, placebo-controlled crossover design was used. Randomization of treatment order was performed using a computer-generated allocation sequence created with the online randomization software Randomizer.org (Figure 1). Each participant completed two experimental sessions within a 10-day period, separated by a minimum washout period of three days to minimize potential carryover effects of nitrate intake. This washout duration was selected based on previous studies demonstrating that plasma nitrate and nitrite concentrations return to baseline within 24–48 h following acute dietary nitrate supplementation, making a three-day interval sufficient to minimize residual physiological effects [14]. Participants were instructed to maintain their habitual diet throughout the study period, except for abstaining from nitrate-rich foods (e.g., beetroot, spinach, arugula, and other leafy green vegetables) during the 72 h preceding each experimental session. Additionally, participants were asked to refrain from strenuous physical activity for 48 h before testing. All experimental sessions were conducted in the morning under standardized fasting conditions. Participants were instructed to arrive at the Laboratory of Biomechanics after an overnight fast and to avoid brushing their teeth, chewing gum, or using antibacterial mouthwash prior to testing to preserve oral nitrate-reducing bacteria involved in the nitrate–nitrite–nitric oxide pathway.

### 2.3. Intervention Protocol

Upon arrival at the laboratory, anthropometric measurements were obtained. Participants then consumed either a nitrate-rich beetroot juice supplement (NO) or a placebo (PLA) according to the randomized allocation sequence. The nitrate-rich beverage consisted of 800 mg of nitrate provided as sodium nitrate together with 5 mL of concentrated beetroot juice diluted in approximately 150 mL of water. The placebo beverage consisted of 5 mL of the same concentrated beetroot juice diluted in approximately 150 mL of water without the addition of sodium nitrate. The placebo beverage contained approximately 57 mg of naturally occurring nitrate. Both beverages were matched for appearance, taste, and volume. The selected dose was based on previous evidence demonstrating ergogenic effects of acute dietary nitrate supplementation in exercise settings. Following supplementation, participants remained seated for a 2 h absorption period before testing. This interval was selected because peak plasma nitrate and nitrite concentrations are typically observed approximately 2 h after ingestion [13,14]. Both participants and investigators involved in data collection remained blinded to the supplementation condition throughout the study.

### 2.4. EMG

Prior to isokinetic testing, self-adhesive Ag/AgCl electrodes (SORIMEX, Toruń, Poland, 1 cm in diameter) for surface electromyography (sEMG) were placed in the bipolar configuration, parallel to the muscle fibres over the vastus lateralis and vastus medialis muscles of the dominant lower extremity in accordance with the SENIAM (Surface Electromyography for the Non-Invasive Assessment of Muscles) guidelines [15]. The distance between the electrodes was 2 cm. Skin preparation included shaving, light abrasion, and cleaning with alcohol to reduce skin impedance. The correctness of electrodes placement was verified by observation of a raw EMG signal during muscle testing. The electromyographic signal was recorded with the Telemyo 2400T G2 device (Noraxon, Scottsdale, AZ, USA). Signal processing was performed with the MyoResearch XP 1.07 Master Edition software. The electromyographic signal was sampled at a frequency of 1000 Hz and filtered with a hardware bandpass filter (bandwidth: 10–500 Hz). In order to obtain muscle fatigue characteristics, two maximum voluntary contraction (MVC) tests were conducted. The first MVC test was performed before isokinetic testing. Participants performed three repetitions of maximal static contraction of the knee extensors with the knee flexed at 90°. Each contraction was held for 5 s and there was a 30 s rest between repetitions. The second MVC test was conducted directly after the isokinetic test, and it consisted of one repetition of maximum static contraction of the knee extensors held for 5 s with the knee flexed at 90°. Before each MVC test, signal quality was verified and the recordings were visually inspected for movement artefacts and possible electrode displacement. The muscle fatigue characteristics consisted of four parameters: mean amplitude, maximum amplitude, mean frequency and median frequency of the electromyographic signal [16] during a 1 s interval taken from first and second MVC testing. For each of the first and second MVC tests, the 1 s interval with the highest mean amplitude was selected. Increase in amplitude parameters as well as decrease in mean or median frequency after fatigue such as isokinetic testing has been observed [16,17]. For the amplitude parameters the electromyographic signal was full-wave-rectified and smoothed using a root mean square algorithm (RMS) with a 50 ms window. The frequency parameters were obtained from the raw signal. Each electromyographic parameter was analyzed by comparing the electromyographic signal from a 1 s interval from the second MVC test [MVC2] with the corresponding 1 s interval from the first MVC test [MVC1]. The formula was as follows:

MVC2/MVC1

A ratio equal to 1 indicated no change between the first (MVC1) and second (MVC2) measurements. For amplitude parameters, values > 1 indicated increased electromyographic amplitude following the isokinetic protocol, whereas values < 1 indicated decreased amplitude. For frequency parameters, values < 1 indicated a reduction in electromyographic frequency following exercise, which is consistent with greater muscle fatigue, whereas values > 1 indicated higher post-exercise frequencies.

The rationale for using the MVC2/MVC1 ratio was to normalize the post-exercise EMG parameters to the individual baseline (MVC1), thereby allowing for the assessment of fatigue-related changes while reducing inter-individual variability. Based on previous studies, muscle fatigue was expected to be characterized by higher mean and maximum amplitude together with lower mean and median frequency during MVC2 compared with MVC1 [16,17].

### 2.5. Lactate Assessment

Capillary blood samples were collected from the fingertip to determine blood lactate concentration. Samples were obtained before the warm-up as the time point before exercise (t0) and again 3 min after completion of the isokinetic fatigue protocol. This post-exercise time point (t1) was selected because blood lactate concentration typically peaks within 2–5 min following high-intensity exercise [18].

Blood lactate concentration was analyzed using a Biosen C-Line analyzer (EKF Diagnostics, Barleben, Germany) according to the manufacturer’s instructions. To ensure consistency, all samples were collected by the same investigator, and the analyzer was calibrated before each testing session following the manufacturer’s recommendations.

### 2.6. Isokinetic Testing Protocol

Prior to the experimental sessions, all participants were familiarized with the testing procedures to ensure consistent execution of the isokinetic tasks. Each testing session began with a standardized 10 min warm-up consisting of low-intensity cycling and dynamic lower-limb exercises designed to prepare the knee extensors and flexors for maximal effort contractions. Following the warm-up, isokinetic testing was performed using a Biodex System 3 dynamometer (Biodex Medical Systems, Shirley, NY, USA). Participants were seated and secured using straps across the trunk and pelvis to minimize extraneous body movement. The dynamometer axis was aligned with the lateral femoral epicondyle of the dominant lower extremity according to the manufacturer’s guidelines. Participants performed concentric knee extension and flexion contractions at angular velocities of 60°/s, 180°/s, and 240°/s. Rest intervals between velocities lasted 60 s. Three maximal repetitions were completed at 60°/s and 180°/s, whereas the 240°/s condition consisted of 30 consecutive repetitions designed to induce neuromuscular fatigue. Previous research demonstrates that high-repetition, high-velocity isokinetic protocols (e.g., 25 repetitions at 240°/s or 30 repetitions at 300°/s) are valid and widely used methods to induce substantial neuromuscular fatigue and to quantify knee muscle endurance through declines in peak torque, work, and power, as well as associated cardiopulmonary responses, thereby supporting the use of a 30-repetition protocol at 240°/s for fatigue assessment in the present study [19,20,21,22] The testing order was selected (180°/s, 60°/s, and 240°/s) to minimize fatigue-induced interference with maximal strength measurements. Strong verbal encouragement was provided throughout all trials to ensure maximal effort.

### 2.7. Statistical Analysis

Statistical analyses were performed using Jamovi (version 2.6.24). Descriptive statistics are presented as the mean ± standard deviation (SD). Data normality was assessed using the Shapiro–Wilk test. Differences in isokinetic performance variables and electromyographic activity between the nitrate (NO) and placebo (PLA) conditions were analyzed using paired samples *t*-test. For blood lactate concentration, repeated-measures analysis of variance (ANOVA) was applied to assess differences across experimental conditions and measurement time points (2 [NO or PLA] × 2 [t0 or t1]). Sphericity was determined using the Mauchly test. The Bonferroni correction was used to compare within-subjects factors. According to the Cohen guidelines related to the effect size, values of Cohen’s *d* were small (0.2), medium (0.5) and large (0.8) for *t*-test, whereas values of the partial eta-squared (η2) were small (0.01), medium (0.06) and large (0.14) for ANOVA [23]. A 95% confidence interval (CI) was calculated. Statistical significance was set at *p* < 0.05. The predefined primary outcomes were peak torque and relative peak power. All remaining isokinetic variables, electromyographic parameters, and post-exercise blood lactate concentration were considered secondary outcomes.

## 3. Results

All variables met the assumption of normality according to the Shapiro–Wilk test (*p* > 0.05). Parametric analyses were therefore applied. All randomized participants completed both experimental conditions, and no participants were lost to follow-up or excluded from the final analyses (n = 13). The predefined primary outcomes (peak torque and relative peak power) are presented first, followed by the secondary isokinetic, electromyographic, and metabolic outcomes.

### 3.1. Primary Outcomes

#### 3.1.1. Peak Torque

**Figure 2:**
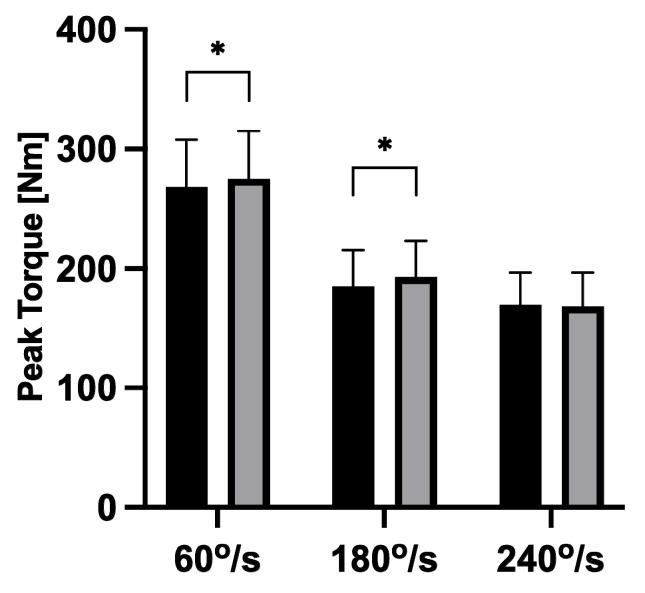
The mean ± SD values of peak torque of contraction during isokinetic testing at 60°/s, 180°/s, and 240°/s under the NO (grey bars) and PLA (black bars) conditions. * Indicates a significant difference between the NO and PLA conditions (*p* < 0.05). NO = nitrate supplementation; PLA = placebo.

Peak torque was significantly greater following nitrate supplementation at 60°/s and 180°/s, whereas no significant difference was observed during the 240°/s fatigue protocol (Table 1, Figure 2).

#### 3.1.2. Relative Power

**Figure 3:**
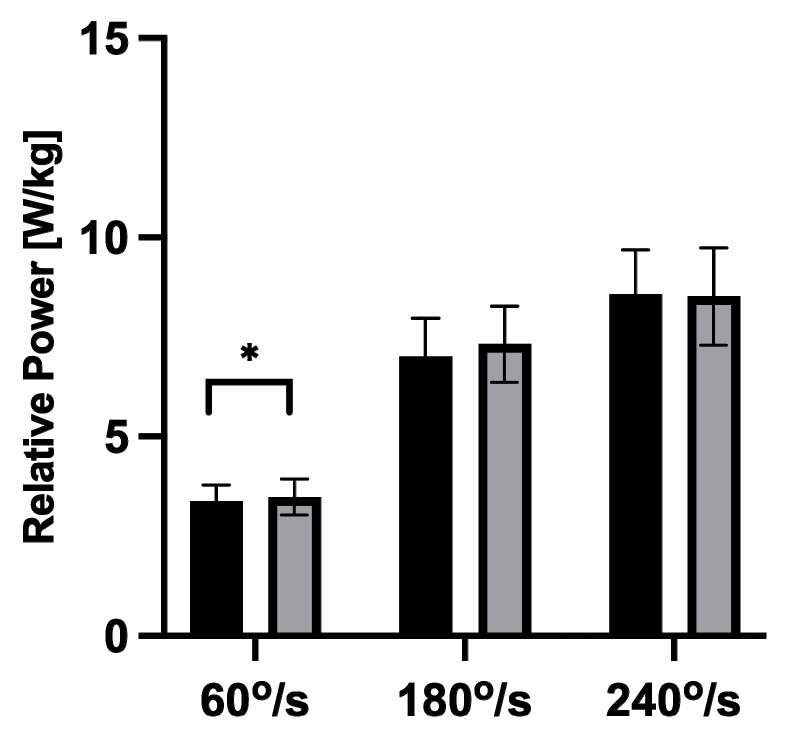
The mean ± SD values of relative power during isokinetic testing at 60°/s, 180°/s, and 240°/s under the NO (grey bars) and PLA (black bars) conditions. * Indicates a significant difference between the NO and PLA conditions (*p* < 0.05). NO = nitrate supplementation; PLA = placebo.

Relative peak power was significantly greater following nitrate supplementation at 60°/s. At 180°/s, despite the *p*-value being borderline, the difference was statistically non-significant. For the 240°/s fatigue protocol, a non-significant difference was also found (Table 1, Figure 3).

### 3.2. Secondary Outcomes

#### 3.2.1. Maximum Repetition Work

**Figure 4:**
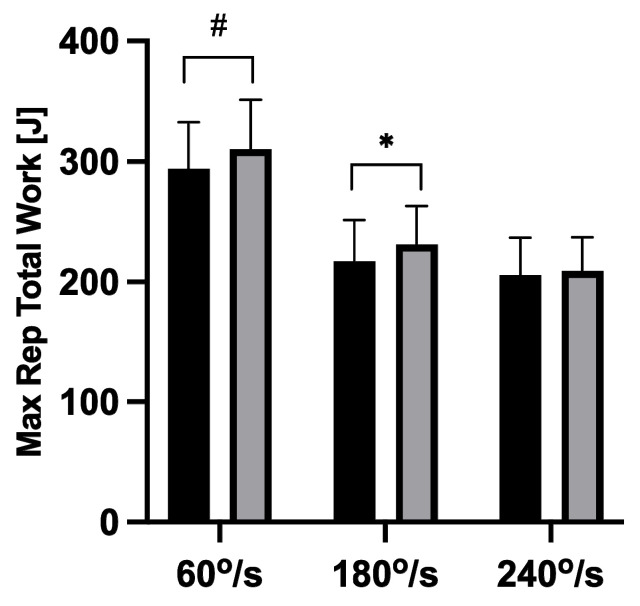
The mean ± SD values of maximum work performed during a single repetition during isokinetic testing at 60°/s, 180°/s, and 240°/s under the NO (grey bars) and PLA (black bars) conditions. * Indicates a significant difference between the NO and PLA conditions (*p* < 0.05), # (*p* < 0.01). NO = nitrate supplementation; PLA = placebo.

Maximum repetition work was significantly greater following nitrate ingestion at both 60°/s (311 ± 40.5 vs. 294 ± 38.3 J; t (12) = −3.39, *p* = 0.005, d = 0.94) and 180°/s (231 ± 32.0 vs. 217 ± 34.3 J; t (12) = −2.49, *p* = 0.029, d = 0.69). No significant differences were observed at 240°/s (*p* = 0.513) (Figure 4).

#### 3.2.2. Torque at 0.18 s

**Figure 5:**
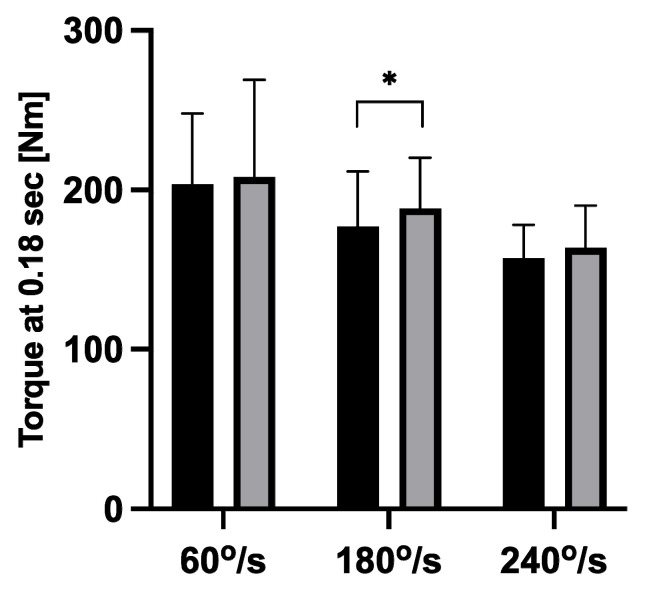
The mean ± SD values of torque at 0.18 s of contraction during isokinetic testing at 60°/s, 180°/s, and 240°/s under the NO (grey bars) and PLA (black bars) conditions. * Indicates a significant difference between the NO and PLA conditions (*p* < 0.05). NO = nitrate supplementation; PLA = placebo.

Torque measured at 0.18 s of knee extension was significantly higher in the NO condition at 180°/s (189 ± 31.5 vs. 177 ± 34.4 Nm; t (12) = −2.28, *p* = 0.042, d = 0.63). No significant differences were observed at 60°/s (*p* = 0.713) or 240°/s (*p* = 0.389) (Figure 5).

#### 3.2.3. Torque at 30°

**Figure 6:**
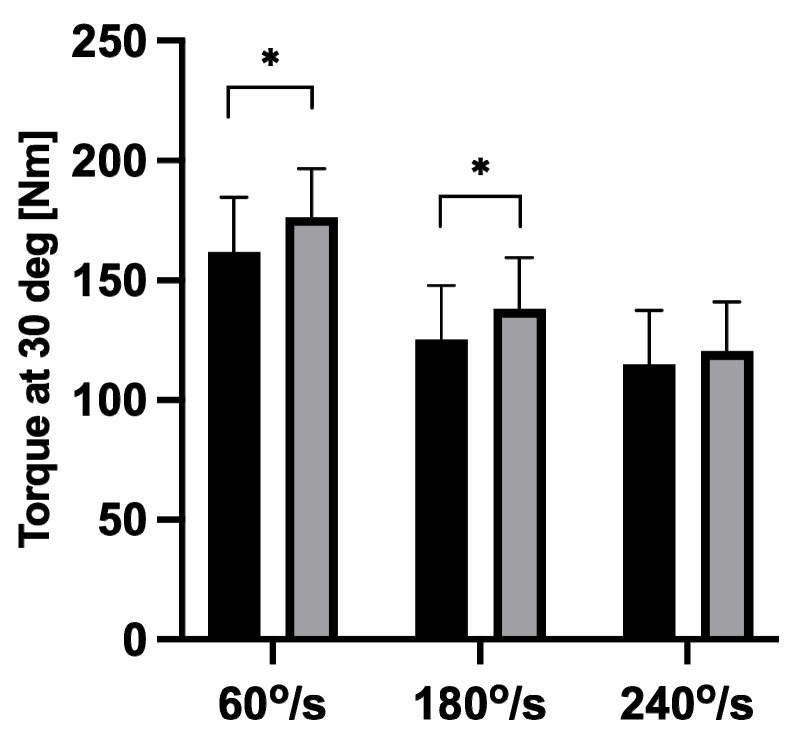
The mean ± SD values of torque at 30 degrees of contraction during isokinetic testing at 60°/s, 180°/s, and 240°/s under the NO (grey bars) and PLA (black bars) conditions. * Indicates a significant difference between the NO and PLA conditions (*p* < 0.05). NO = nitrate supplementation; PLA = placebo.

Torque measured at 30° of knee extension was significantly greater following nitrate ingestion at both 60°/s (176 ± 20.2 vs. 162 ± 22.9 Nm; t(12) = −2.47, *p* = 0.029, d = 0.69) and 180°/s (138 ± 21.5 vs. 125 ± 22.6 Nm; t (12) = −2.52, *p* = 0.027, d = 0.70). No significant differences were observed at 240°/s (*p* = 0.267) (Figure 6).

### 3.3. Electromyographic Activity

**Figure 7:**
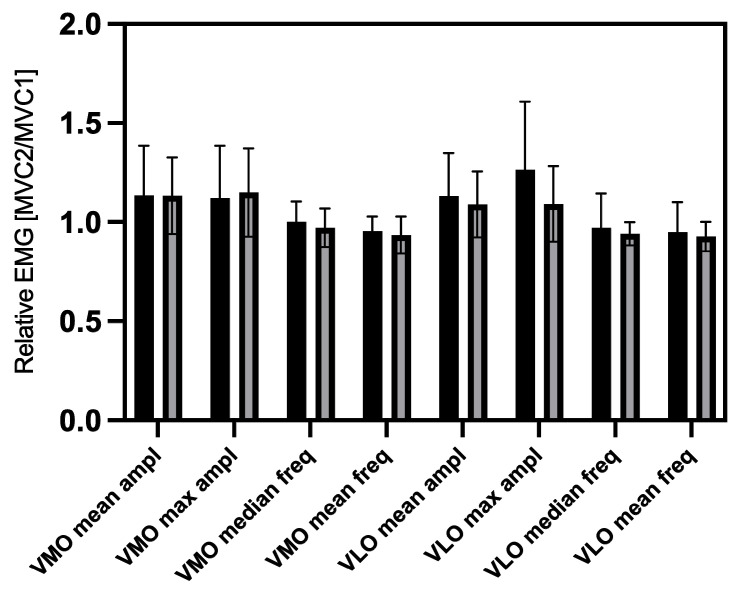
The mean ± SD values of EMG activity during the MVC_2_ relative to the MVC_1_ under the NO (grey bars) and PLA (black bars) conditions. NO = nitrate supplementation; PLA = placebo.

Electromyographic activity of the vastus medialis oblique (VMO) and vastus lateralis oblique (VLO) muscles was analyzed in terms of normalized signal amplitude and frequency characteristics (Figure 7). No significant differences between the NO and PLA conditions were observed for mean EMG amplitude in the VMO muscle (1.133 ± 0.193 vs. 1.134 ± 0.252; *p* = 0.991) or maximal EMG amplitude (1.149 ± 0.224 vs. 1.122 ± 0.264; *p* = 0.724). Similarly, no significant differences were detected for median frequency (0.972 ± 0.097 vs. 1.002 ± 0.103; *p* = 0.305) or mean frequency (0.935 ± 0.094 vs. 0.955 ± 0.074; *p* = 0.443). For the VLO muscle, no significant differences were observed in mean EMG amplitude between NO and PLA conditions (1.089 ± 0.167 vs. 1.131 ± 0.219; *p*= 0.512). Maximal amplitude also did not differ significantly between conditions (1.092 ± 0.191 vs. 1.265 ± 0.343; *p* = 0.106). Similarly, frequency domain parameters for the VLO muscle were not significantly affected by nitrate supplementation. The median frequency (0.941 ± 0.058 vs. 0.972 ± 0.173; *p* = 0.605) and mean frequency (0.927 ± 0.074 vs. 0.950 ± 0.150; *p* = 0.643) were comparable between experimental conditions.

### 3.4. Blood Lactate Concentration

**Figure 8:**
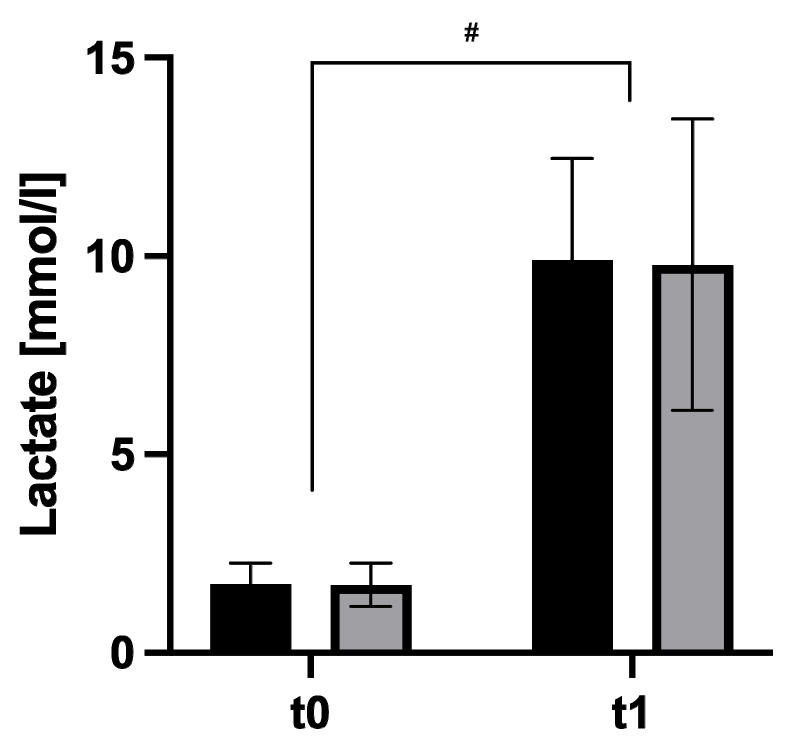
The mean ± SD values of blood lactate concentration measured before exercise and 3 min after completion of the fatigue protocol under the NO (grey bars) and PLA (black bars) conditions. # Significantly different from pre-exercise values (*p* < 0.01). NO = nitrate supplementation; PLA = placebo; t0 = time point before exercise; t1 = time point after completion of the isokinetic fatigue protocol.

Blood lactate concentration was analyzed using a repeated-measures ANOVA with supplementation condition (nitrate vs. placebo) and time as within-subject factors. The results of the Mauchly test (W = 1.0, *p* > 0.05) indicated that the condition of sphericity was fulfilled. Blood lactate concentrations increased from 1.71 ± 0.551 to 9.78 ± 3.68 mmol/L in the nitrate condition and from 1.73 ± 0.523 to 9.90 ± 2.56 mmol/L in the placebo condition. Accordingly, a significant main effect of time was observed (F (1,12) = 93.66, *p* = 0.001, η^2^ = 0.775) (Figure 8), indicating a substantial increase in lactate concentration following the isokinetic exercise protocol. However, no significant main effect of supplementation was found (F (1,12) = 0.08, *p* = 0.780), suggesting that nitrate supplementation did not significantly influence blood lactate levels. Additionally, no significant interaction between time and supplementation condition was observed (F (1,12) = 0.04, *p* = 0.853).

## 4. Discussion

The primary finding of this study is that acute dietary nitrate supplementation improved selected isokinetic performance variables during low- and moderate-velocity contractions. A single dose of nitrate significantly enhanced peak torque and selected power-related variables at 60°/s and 180°/s, whereas no significant effects were observed at 240°/s.

However, the 240°/s condition was assessed using a 30-repetition fatigue protocol. Therefore, the absence of significant effects at this angular velocity should be interpreted with caution, as the present design does not allow the effects of contraction velocity to be fully distinguished from those of the fatigue protocol.

The improvements in peak torque observed at 60°/s and 180°/s, together with the increase in relative peak power at 60°/s, are consistent with the hypothesis that nitrate supplementation may enhance the force-generating capacity of skeletal muscle. Dietary nitrate increases nitric oxide (NO) bioavailability through the sequential reduction of nitrate to nitrite and subsequently to NO [1,24,25]. Previous experimental studies suggest that nitric oxide may influence muscle contractility by modulating calcium (Ca^2+^) handling within the sarcoplasmic reticulum and improving excitation–contraction coupling [25,26,27,28]. Although these mechanisms provide a plausible explanation for the present findings, they were not directly assessed in this study. The present findings add to the current understanding of the ergogenic effects of dietary nitrate under different isokinetic testing conditions. Previous studies, including Coggan et al. [8], reported that the ergogenic effects of dietary nitrate were most pronounced during high-velocity contractions, which was attributed to the preferential influence of nitric oxide on type II muscle fibres. In contrast, the present study demonstrated significant improvements in peak torque and relative peak power during the 60°/s and 180°/s protocols, whereas no significant effects were observed at 240°/s. This apparent discrepancy may be related to methodological differences between the studies. Nevertheless, the findings suggest that acute dietary nitrate supplementation may enhance muscle performance under selected isokinetic testing conditions. In line with previous observations, Coggan et al. [29] proposed that increased nitric oxide availability may improve the contractile properties of skeletal muscle. Although the present study did not directly assess these physiological mechanisms, they may provide one possible explanation for the improvements observed in peak torque and relative peak power.

The present findings are also generally consistent with the recent observations of Daneshparvar et al. [9], who reported improvements in selected isokinetic performance variables following acute beetroot juice supplementation. Although methodological differences between the studies should be acknowledged, both investigations support the potential ergogenic effect of dietary nitrate on selected measures of isokinetic muscle performance. These observations are also supported by the recent findings of Eroglu et al. [10], who demonstrated increased peak and mean power following acute beetroot juice supplementation during a Wingate test in trained football players. Although a different exercise modality was employed, both studies indicate that acute dietary nitrate supplementation may enhance high-intensity muscle performance.

Further support for a potential effect of dietary nitrate on muscle contractile performance comes from the recent study by Wei et al. [30]. The authors reported that lower nitrate doses improved the rate of torque development, whereas higher doses were required to increase maximal torque production. Although the rate of torque development was not assessed in the present study, these observations are compatible with the possibility that dietary nitrate may influence force production characteristics. However, direct comparisons should be made with caution because different performance outcomes and experimental protocols were evaluated. Moreover, the observed improvements in torque angle characteristics may reflect enhanced torque production during critical phases of the movement.

An important observation of the present study is the absence of significant differences in EMG amplitude and frequency parameters of the VMO and VLO muscles between nitrate and placebo conditions. These findings indicate that nitrate supplementation did not significantly modify the pre- to post-exercise changes in the EMG parameters obtained during maximal voluntary contractions (MVCs). Similar findings have been reported in previous studies that demonstrated no significant effects of nitrate supplementation on voluntary muscle activation [30,31]. However, because EMG was assessed during MVC tests performed before and after the isokinetic protocol rather than during the dynamic isokinetic contractions themselves, the present study cannot determine whether nitrate supplementation influenced neuromuscular recruitment during dynamic exercise. Therefore, the combination of improved mechanical performance variables with unchanged MVC-derived EMG parameters is consistent with, but does not demonstrate, enhanced efficiency of force production at the muscular level rather than altered neural drive [29,30]. No significant differences in post-exercise blood lactate concentration were observed between experimental conditions. Therefore, the present findings do not indicate that nitrate supplementation altered the overall post-exercise lactate response. However, because blood lactate was assessed only before exercise and three minutes after completion of the fatigue protocol, no conclusions can be drawn regarding lactate kinetics or velocity-specific metabolic responses. Interestingly, Eroglu et al. [10] reported significantly higher post-exercise blood lactate concentrations following acute beetroot juice supplementation despite improvements in peak and mean anaerobic power during a Wingate test. This contrasts with the present findings and may reflect differences in exercise modality, exercise duration, participant characteristics, and the metabolic demands of the testing protocols. Previous studies have suggested that nitrate supplementation may improve metabolic efficiency, potentially through reductions in the ATP cost of force production or enhanced mitochondrial efficiency [5,32,33]. Whether these mechanisms contributed to the present findings cannot be determined from the current data.

The absence of significant effects during the 240°/s fatigue protocol may reflect several factors. Previous studies have suggested that fatigue-related metabolite accumulation, disturbances in intracellular homeostasis, and impaired excitation–contraction coupling may reduce muscle force production during repeated maximal efforts [25,30,31]. However, because these variables were not directly assessed in the present study, their contribution to the observed findings remains speculative. Under these conditions, disturbances in intracellular homeostasis may mask the potential ergogenic effects associated with increased nitric oxide bioavailability [34]. Second, at high angular velocities, the time available for force generation is substantially reduced, potentially limiting the expression of differences in maximal torque and power output. Finally, under the experimental conditions of the present study, nitrate supplementation appeared to be more effective in the 60°/s and 180°/s maximal contraction protocols than in the 240°/s fatigue-oriented protocol [35,36,37]. This observation differs from previous findings reported by Husmann et al. [38], who demonstrated improved exercise tolerance and reduced muscle fatigue following dietary nitrate supplementation. These discrepancies may reflect differences in exercise modality, outcome measures, and the physiological demands of the testing protocols. Although plasma nitrate/nitrite concentrations were not assessed in the present study, the supplementation protocol was identical to that previously shown [13] to markedly elevate circulating NOx concentrations within the same time frame. Taken together, these observations are consistent with the possibility that the observed improvements originated predominantly from peripheral adaptations previously proposed to be associated with nitrate supplementation. However, this interpretation should be considered speculative because plasma nitrate/nitrite concentrations and the proposed physiological mechanisms were not directly assessed in the present study.

From a practical perspective, the improvements observed in peak torque and relative peak power were modest, corresponding to approximately 3–4% compared with placebo. Although these changes may appear small, improvements of this magnitude could be meaningful in sports requiring repeated maximal force production or explosive lower-limb actions, where relatively small changes in muscle performance may contribute to competitive performance. However, the present findings were obtained under controlled laboratory conditions using isokinetic dynamometry, and their translation to sport-specific performance or functional outcomes requires further investigation. Therefore, the practical significance of these findings should be interpreted with caution until confirmed in larger studies and in applied athletic settings.

Several limitations of the present study should be acknowledged. First, plasma nitrate and nitrite concentrations were not measured; therefore, the physiological response to supplementation and the extent of nitric oxide bioavailability could not be directly confirmed. In addition, the placebo beverage contained a small amount of naturally occurring nig nitrate rather than being completely nitrate-free. Therefore, a minor physiological effect of the placebo intervention cannot be completely excluded. Second, the relatively small sample size may have limited the statistical power to detect smaller effects, particularly during the fatigue protocol. In addition, although the sample size calculation was based on the expected physiological response to dietary nitrate supplementation, future studies should consider powering the analysis using the primary functional performance outcomes. Third, the study included only physically active young men, which limits the generalizability of the findings to women, older adults, and other athletic populations. In addition, blood lactate concentration was assessed at only two time points (pre- and post-exercise), preventing a more detailed evaluation of lactate kinetics. Furthermore, although peak torque and relative peak power were predefined as the primary outcomes, multiple secondary outcomes were analyzed without adjustment for multiple comparisons. Therefore, these exploratory findings should be interpreted with appropriate caution. Although participants received standardized dietary instructions regarding nitrate-rich foods, adherence was not objectively verified using dietary records or food diaries. Therefore, the potential influence of habitual dietary nitrate intake cannot be completely excluded. Finally, surface electromyography was recorded only from the vastus medialis oblique and vastus lateralis oblique muscles. Therefore, the present EMG analysis may not fully represent the neuromuscular activity of all muscles contributing to the tested movements. Future studies involving larger and more diverse populations are warranted to confirm the present findings. Simultaneous assessment of plasma nitrate/nitrite concentrations and additional physiological markers may further clarify the mechanisms underlying the ergogenic effects of dietary nitrate supplementation. Moreover, dose–response studies and investigations using different isokinetic testing protocols may help establish the optimal supplementation strategy and identify the exercise conditions under which nitrate supplementation is most effective.

## 5. Conclusions

In conclusion, the present findings suggest that acute dietary nitrate supplementation may improve selected isokinetic performance variables, including peak torque and selected power-related variables, during the 60°/s and 180°/s protocols. No significant effects were observed during the 240°/s condition. Because the 240°/s condition consisted of a fatigue-oriented protocol, the present study cannot determine whether the absence of significant effects was attributable to contraction velocity, fatigue, or the combined influence of both factors. The findings should be interpreted as preliminary considering the relatively small sample size, the exploratory analysis of multiple secondary outcomes, and the absence of direct verification of nitrate/nitrite bioavailability. Further studies involving larger cohorts, direct assessment of physiological mechanisms and both male and female participants are warranted to confirm and extend these findings and to determine potential sex related differences in the ergogenic response to acute dietary nitrate ingestion.

## Figures and Tables

**Table 1:** Predefined primary outcomes following nitrate (NO) and placebo (PLA) supplementation. Data are presented as the mean ± SD. Mean percentage difference was calculated as (NO − PLA) ×100/max(NO; PLA). Cohen’s *d* represents the standardized effect size.

Outcome	Velocity (°/s)	PLA	NO	Difference (%)	Cohen’s *d*	95% CI	*p*
Peak Torque (Nm)	60	268.2 ± 39.8	275.3 ± 39.9	2.6	0.6	0.0–1.2	0.045
	180	185 ± 30.6	193.1 ± 30.2	4.2	0.6	0.0–1.2	0.049
	240	169.7 ± 27.2	168.5 ± 28.2	−0.7	−0.1	−0.6–0.5	0.723
Relative Peak Power (W/kg)	60	3.4 ± 0.4	3.5 ±0.5	2.9	0.7	0.1–1.3	0.030
	180	7 ± 1.0	7.3 ± 1.0	4.1	0.6	0.0–1.2	0.050
	240	8.6 ± 0.3	8.5 ± 0.3	−1.2	−0.1	−0.6–0.5	0.768

Notes: NO—nitrate, PLA—placebo, CI—confidence interval.

## Data Availability

The data presented in this study are available upon request from the corresponding author due to privacy and ethical restrictions.

## Abbreviations

EMG	electromyography
NO	nitric oxide
MVC	maximal voluntary contraction
PLA	placebo
VLO	vastus lateralis oblique
VMO	vastus medialis oblique
